# Evaluation of Various Types of Alginate Inks for Light-Mediated Extrusion 3D Printing

**DOI:** 10.3390/polym16070986

**Published:** 2024-04-04

**Authors:** Aitana Zoco de la Fuente, Ane García-García, Leyre Pérez-Álvarez, Isabel Moreno-Benítez, Asier Larrea-Sebal, Cesar Martin, Jose Luis Vilas-Vilela

**Affiliations:** 1 Macromolecular Chemistry Group (LABQUIMAC), Physical Chemistry Department, Faculty of Science and Technology, University of the Basque Country UPV/EHU, 48940 Leioa, Spain; aitanazoco@gmail.com (A.Z.d.l.F.); ane.garcia@bcmaterials.net (A.G.-G.); joseluis.vilas@ehu.eus (J.L.V.-V.); 2 BCMaterials, Basque Center for Materials, Applications and Nanostructures, UPV/EHU Science Park, 48940 Leioa, Spain; 3 Macromolecular Chemistry Group (LABQUIMAC), Organic Chemistry Department, Faculty of Science and Technology, University of the Basque CountryUPV/EHU, 48940 Leioa, Spain; misabel.moreno@ehu.eus; 4 Biofisika Institute (UPV/EHU, CSIC), UPV/EHU Science Park, 48940 Leioa, Spain; asier.larrea@ehu.eus (A.L.-S.); cesar.martin@ehu.eus (C.M.); 5 Department of Biochemistry and Molecular Biology, Faculty of Science and Technology, University of the Basque Country UPV/EHU, 48940 Leioa, Spain; 6 Fundación Biofisika Bizkaia, Barrio Sarriena s/n, 48940 Leioa, Spain

**Keywords:** methacrylated alginate, photo-cross-linking, extrusion printing

## Abstract

Naturally derived biopolymers modifying or combining with other components are excellent candidates to promote the full potential of additive manufacturing in biomedicine, cosmetics, and the food industry. This work aims to develop new photo-cross-linkable alginate-based inks for extrusion 3D printing. Specifically, this work is focused on the effect of the addition of cross-linkers with different chemical structures (polyethylene glycol diacrylate (PEGDA), *N,N′*-methylenebisacrylamide (NMBA), and acrylic acid (AA)) in the potential printability and physical properties of methacrylated alginate (AlgMe) hydrogels. Although all inks showed maximum photo-curing conversions and gelation times less than 2 min, only those structures printed with the inks incorporating cross-linking agents with flexible and long chain structure (PEGDA and AA) displayed acceptable size accuracy (~0.4–0.5) and printing index (Pr ~1.00). The addition of these cross-linking agents leads to higher Young’s moduli (from 1.6 to 2.0–2.6 KPa) in the hydrogels, and their different chemical structures results in variations in their mechanical and rheological properties. However, similar swelling ability (~15 swelling factor), degradability (~45 days 100% weight loss), and cytocompatibility (~100%) were assessed in all the systems, which is of great importance for the final applicability of these hydrogels.

## 1. Introduction

Three-dimensional (3D) printing is a growing technology that could be considered the future universal mode of manufacturing in different industries, e.g., biomedicine, tissue engineering, food, or pharmaceuticals [1,2,3,4,5]. This layer-by-layer construction technology has also been incorporated over the last few years in the fabrication of hydrogels [6]. The strategy behind the 3D printing of hydrogels is that the ink remains in the liquid state and undergoes sol–gel transition through the application of a specific stimulus. In this regard, thermoinduced, pH/ion-induced, and photoinduced methods can be differentiated. In the case of photoinduced hydrogels, they remain in a liquid state before the light source is applied, and when they are exposed to UV or visible light with a specific wavelength, they can be cured in situ and become photopolymerized/photo-cross-linked hydrogels [7]. According to the delivery mode, extrusion-mediated printing, due to its simplicity, is one of the most relevant 3D printing techniques that employs photo-cross-linkable inks [8].

Extrusion-based 3D printing is founded on a dispensing head that moves in an automatically controlled way and allows a continuous deposition of the ink onto a surface according to a previously designed digital pattern.

Undoubtedly, due to their chemical and physical properties, hydrogels are the best candidates for printable materials in both the biomedicine and food industries. On the one hand, they resemble soft, living tissue, and on the other hand, they endow food with distinctive textures, allowing them to act as active matrices for nutrients [9].

In this context, the development of hydrogel-based inks obtained from renewable sources is a current challenge. In this sense, there are many biopolymers to be considered as hydrogel-forming inks for 3D printing. This is the case for algae-based materials such as alginate, which is one of the most widely studied biopolymers due to its ease of chemical functionalization and biological properties. Alginate, composed of (1–4)-linked β-D-mannuronic (M) and α-L-guluronic (G) acids, is typically used in the form of hydrogel in biomedicine and the food industry [10]. Chemical and/or physical cross-linking has been exploited for the extrusion-mediated 3D printing of alginate. In the case of physical cross-linking, the most common printing methodology is based on the association of alginate G units with divalent cations [11]. However, one critical drawback of ionically cross-linked alginate gels is their limited long-term stability due to exchange reactions with monovalent cations and polyanions [12]. Consequently, a growing interest in covalently cross-linked alginate hydrogels has developed.

Photo-cross-linking is an effective method to prepare covalent networks of alginate, but requires its prior modification of alginate to incorporate functional groups such as acrylates or methacrylates. This functionalization can be achieved with the addition of 2-aminoethylmethacrylate hydrochloride [13,14,15,16,17,18], glycidyl methacrylate [13,15], or methacrylic anhydride [15,19,20,21,22,23,24]. The incorporation of those groups allows free radical cross-linking in the presence of a photoinitiator and after exposure to low light. It has been demonstrated that by varying the UV exposure time or the photoinitiator, the mechanical properties and the swelling of alginate hydrogels can be regulated, which is of great importance in further application [14,25].

Along these lines, research has focused on employing external cross-linking agents in methacrylated alginate formulations, such as other acrylated biopolymers [12,15,26] or synthetic polyethylene glycol diacrylate (PEGDA) molecules [27,28]. Indeed, PEGDA polymer has demonstrated successful photo-curing and enhanced mechanical properties when it is combined with methacrylated hydrogels [29]. Nevertheless, at this point, investigations have generally focused on the addition of methacrylated alginate as a cross-linking agent in PEG-acrylated networks [27,30]. However, even given the extent of investigations into photo-cross-linkable alginate inks, the accurate 3D printing of stable structures based on alginate remains a challenging issue that is still in its early stages of exploration.

Taking this into account, the present work aims to analyze the possibilities of different cross-linking molecules to be included in methacrylated alginate ink formulations for extrusion-based, light-mediated 3D printing. To this end, polyethylene glycol diacrylate is compared with a short-chain cross-linker typically employed in hydrogel formulations, *N*,*N*’-methylenebisacrylamide (NMBA), in order to analyze the effect of the chemical structure of the cross-linker on the printability of the ink. On the other hand, the polymerization of acrylic acid (AA) in the presence of methacrylated alginate leads to interpenetrating networks in which carboxylic groups of AA can develop intermolecular interactions, such as hydrogen bonds and dipole–ion, with alginate [31]. Accordingly, in this work, AA is for the first time explored as a cross-linking agent for methacrylated alginate ink. Herein, the printability of a series of three inks was analyzed and compared with pristine methacrylated alginate. Moreover, the mechanical and rheological properties, swelling capacity, degradation profile, and cytotoxicity of printed hydrogels were also studied.

## 2. Materials and Methods

### 2.1. Materials

The synthesis of alginate-based hydrogels was performed by modifying sodium alginate (Sigma-Aldrich, St. Louis, MO, USA,) with methacrylic anhydride (C_8_H_10_O_3_, 154.16 g/mol, 94%, Sigma Aldrich). For that, solutions of sodium hydroxide (NaOH, 40 g/mol, Panreac AppliChem, Barcelona, Spain) and ethanol (C_2_H_5_OH, 46.07 g/mol, 99.5%, Panreac) were prepared. Lithiumphenyl-2,4,6-trimethylbenzoylphosphinate (LAP) (C_16_H_16_LiO_3_P, 294.21 g/mol, 95.5%, Sigma Aldrich) was used as photoinitiator. Polyethylene glycol diacrylate (PEGDA) (C_3_H_3_O(OCH_2_CH_2_)_n_C_3_H_3_O_2_, Mn 700, Sigma Aldrich), acrylic acid (AA) (C_3_H_4_O_2_, 72.06 g/mol, Fluka^TM^, Charlotte, NC, USA) and *N*,*N*’-methylenebisacrylamide (NMBA) (C_7_H_10_O_2_N_2_, 154.17 g/mol, 99%, Sigma Aldrich) were used as cross-linking agents.

### 2.2. Methacrylation of Alginate

#### 2.2.1. Alginate Methacrylation

Methacrylated alginate (AlgMe) was synthesized following the procedure of Chou et al. [32]. Briefly, a 2% sodium alginate sample was prepared and a 20% molar excess of methacrylic anhydride added while adjusting the pH to 7 using an aqueous solution of NaOH (5 M). The final solution was maintained at 4 °C for 24 h, then poured out and washed with ethanol (30 min × 5) in order to remove excess anhydride. The obtained product was dissolved in distilled water and purified via dialysis against sterile water for 3 days. Finally, the sample was lyophilized at −50 °C and 0.2 mbar.

##### H-NMR

The degree of methacrylation of alginate was determined from ^1^H-NMR spectra. For this, a 1.5% (*w*/*w*) methacrylated alginate solution was prepared in deuterated water. ^1^H-NMR spectra were taken on a Bruker Advance 500 MHz spectrometer at 85 °C. According to the literature [33] and assuming that in alginate only G units can be methacrylated, the G ratio in the alginate chain (G%) and the degree of methacrylation (MD%) were calculated using Equations (1) and (2), respectively. H_G_ and H_M_ represent the integral of the hydrogen of the anomeric carbon of the guluronic and mannuronic units at 4.8–5.2 and 4.4 ppm, respectively, and H_a_ and H_b_ represent the integrals of the two vinyl protons of the methacrylic group (5.5 and 6.5 ppm), respectively [34]:

(1) $$G\;\left(\%\right)=\frac{H_{G}}{H_{M}+H_{G}}\times 100$$

(2) $$MD\left(\%\right)=\frac{\frac{H_{a}+H_{b}}{2}}{H_{G}}\times G$$

Data correspond to the average of three samples.

### 2.3. Printing of the Hydrogels

Synthesized methacrylated alginate (AlgMe) was dissolved (8% (*w*/*w*)) in distilled water. Separately, an LAP photoinitiator (1% (*w*/*w*)) was dissolved in distilled water (300 µL) and added to the mixture. The cross-linking agents PEGDA, NMBA, or acrylic acid (20 mM) were added to the already prepared AlgMe solution. Photo-cross-linking and printing was carried out on an INKREDIBLE+ Cellink bioprinter (Cellink, Gothenburg, Sweeden) equipped with UV light LED operating at 405 nm (4 mW/cm^2^) during the printing process. Gelation time of the inks under the lamp of the INKREDIBLE+ Cellink bioprinter was determined following the so-called inverted tube test as the moment when the solution stopped flowing after inverting the test tube.

#### 2.3.1. Photo-DSC Analysis

The UV curing process was studied by photo-DSC (isothermal mode at 25 °C) using a DSC (TA Instruments Q2000, Waters Corporation, New Castle, DE, USA) equipped with a photo-calorimetric accessory with a 200 W mercury lamp operating in an optical range from 320 to 500 nm and an intensity between 1 and 2 mW/cm^2^. Heat flow was normalized with respect to the highest value in each sample from 0 to 1 mW.

#### 2.3.2. Optimization of the Printing

Two different cartridge nozzles were compared, one with an internal diameter of 0.254 mm and the other with an internal diameter of 0.41 mm. The extrusion pressure (10, 15, 20, 25 kPa) and the printing velocity (300, 500, 600 mm/min) were varied and hydrogel strands were printed three times in order to optimize analyzed printing parameters.

#### 2.3.3. Printing Quality

**Figure 1:**
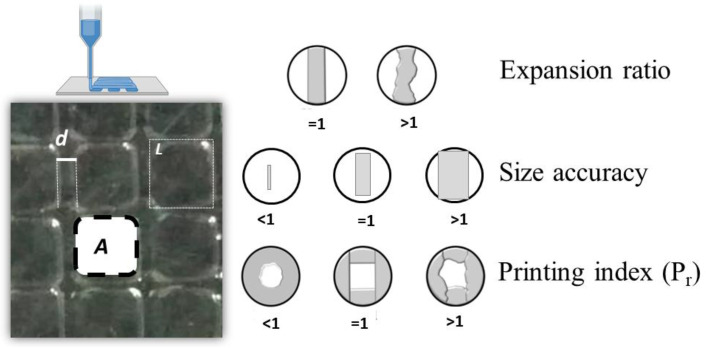
Schematic illustration of the physical representation of the printing quality parameters used.

Using CellInk HeartWare designer software (2.4), apart from strands, square structures of 40 mm × 40 mm were designed (pore dimensions of 5 mm × 5 mm). A Nikon AZ100 Multizoom microscope was employed to obtain images of the printed structures. The printing quality of the inks was analyzed by the processing of images (ImageJ 1.49) of three structures using the comparative parameters (Figure 1) of expansion ratio (α), uniformity factor (U), size accuracy, and printing index (P_r_).

The expansion ratio (Equation (3)) represents the relationship between the diameter of the printed strand (d) and the theoretical diameter of the nozzle (D):

expansion ratio (α) = d/D(3)

Size accuracy was calculated according to Equation (4), in which A_t_ is the theoretical area of the pore and A is that of printed squares:

(4) $$Size\;accuracy=1-\frac{A_{t}-A}{A_{t}}$$

The printing index (P_r_), was calculated following Equation (5) [35], where L is the perimeter of the pore and A is the area:

(5) $$Printing\;index\;(P_{r})=\frac{L^{2}}{16A}$$

### 2.4. Hydrogel Characterization

#### 2.4.1. Mechanical Properties

Compression tests were performed using a Metrotec FTM-50 equipped with a 20 N load cell applying a deformation speed of 1 mm/min. The elastic moduli of the samples (10 mm × 5 mm and 1 mm height) was determined from stress–strain diagrams (12–22% strain). Mean values were calculated from the results obtained for a minimum of 5 different samples.

#### 2.4.2. Rheology

The dynamic rheological behavior of the hydrogels was analyzed in a Rheometric Scientific Advanced Rheometric Expansion System (ARES TA instruments with Peltier oven (APS)) using a parallel plate geometry (25 mm of diameter) with a gap distance of 1 mm. The effect of increasing the angular frequency on the samples was measured in order to evaluate the storage (G’) and loss modulus (G’’). Angular frequencies from 0.1 to 1000 rad/s at a constant strain of 1% were used for all the measurements.

#### 2.4.3. Morphology

The morphology and pore size of the hydrogels were analyzed by scanning electron microscopy (SEM) using a Hitachi S-3400N microscope (150 s, 20 mA, 15 kV, ×50,000 amplification). To this end, lyophilized hydrogels (−50 °C, 0.1 mbar) were coated thinly with gold. Average pore size was calculated for each hydrogel after processing SEM photographs with ImageJ 1.49.

#### 2.4.4. Swelling Behavior

After lyophilization (−50 °C and 0.2 mbar) of printed hydrogels (10 × 5 mm and 1 mm height), they were incubated in phosphate-buffered solution (PBS) (pH = 7.4) at 37 °C. The solution was replaced every day, and at predetermined time points, the samples were removed, rinsed with distilled water, superficially dried and weighed (W_d_). Accordingly, the swelling ratio was measured over time (Equation (6)):

(6) $$Swelling\;factor=\frac{W_{s}-W_{d}}{W_{d}}$$

where W_s_ and W_d_ are the weights of the swollen and dried hydrogels, respectively.

The data correspond to the average of three samples.

### 2.5. In Vitro Degradation

Fresh hydrogels (10 mm × 5 mm and 1 mm height) were incubated in phosphate-buffered solution (PBS) at pH = 7.4 and 37 °C. The mass loss of the hydrogels with time in incubation conditions was registered as a quantification of the in vitro hydrolytic degradation using Equation (7):

(7) $$Mass\;loss\left(\%\right)=\frac{W_{0}-W_{t}}{W_{0}}\times 100$$

where W_0_ is the weight of the hydrogel at initial time and W_t_ at t_∞_. PBS incubation solution was replaced every day, and at certain times, the samples were removed, rinsed with distilled water, superficially dried and weighed. Three samples were evaluated for each data point.

### 2.6. In Vitro Cytotoxicity Essay

The HEK293 cell line (ATCC: HEK-293 CRL-1573 ™) exhibiting epithelial morphology isolated from the kidney of a human embryo was used to measure hydrogel biocompatibility. In sum, 40 × 10^3^ cells were cultured in DMEM supplemented with 10% FBS, 100 µg/mL of streptomycin, 100 U/mL of penicillin, and L-glutamine in 96-well culture plates with or without photo-cross-linking to proliferate for 24 and 48 h. After growth, the cells were rinsed with PBS and fixed using a 4.5% solution of paraformaldehyde in PBS for 30 min at room temperature. Postfixation, the cells were rinsed with PBS and then stained with a 0.5% crystal violet solution for 20 min at room temperature. Following staining, the cells were washed with water. To each well, 200 µL of 15% acetic acid was added, and the plates were shaken for 20 min at room temperature. The acetic acid was then moved to a fresh 96-well plate, and the absorbance was measured at 570 nm using a plate reader. Cell viability percentages were calculated by comparing the absorbance values of cells in the hydrogel wells to those directly seeded in the 96-well plates.

## 3. Results

### 3.1. Alginate Methacrylation

**Figure 2:**
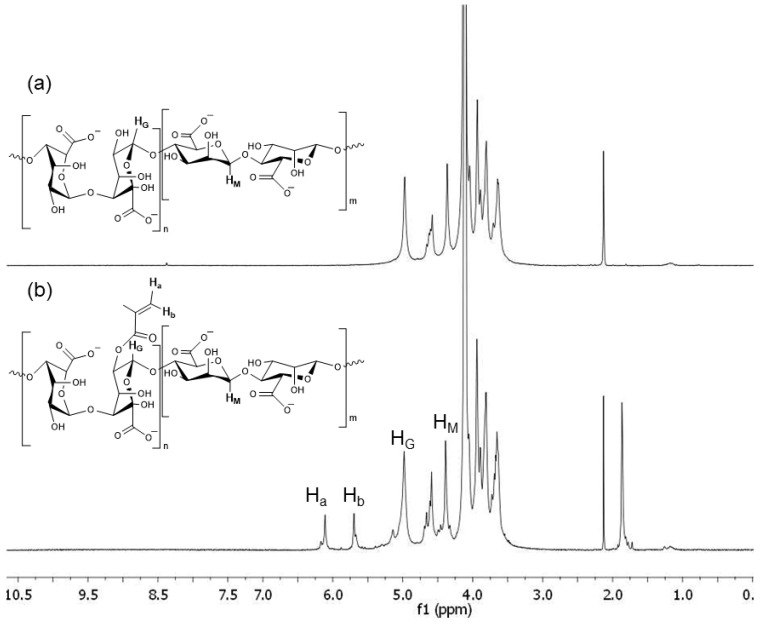
^1^H-NMR spectra of (**a**) alginate and (**b**) methacrylated alginate.

Nuclear magnetic resonance ^1^H-NMR was used to determine the methacrylation degree of the synthesized AlgMe. As expected, all spectra (Figure 2) displayed characteristic peaks between 3.50 and 4.50 ppm, corresponding to the saccharide units of the alginate backbone. In addition, the signals related to the vinyl hydrogens of the methacryloyl group appeared as two well-defined singlets (5.50–6.50 ppm), which are not observed in pure alginate, proving that the modification reaction took place successfully. Meanwhile, the signal of the methyl hydrogens of each methacryloyl group appeared as a well-defined singlet at 2.20 ppm.

The G proportion in the alginate chain and the methacrylation degree (DS) were calculated according to Equations (1) and (2), and was 54.24 ± 0.53% and 8.77 ± 3.54%, respectively.

### 3.2. Extrusion-Mediated Photoprinting of the Hydrogels

**Figure 3:**
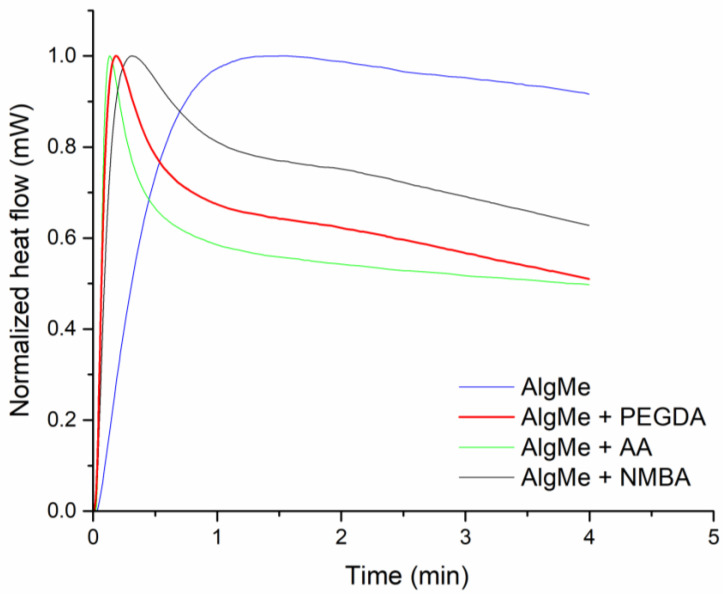
Photo-curing heat flow during photo-DSC measurement of (blue) methacrylated alginate, and methacrylated alginate ink incorporating (red) polyethylene glycol diacrylate, (black) *N*,*N*’-methylenebisacrylamide, and (green) acrylic acid.

Photo-DSC analysis was employed to study the exothermic profile of the photo-cross-linking reaction, which provides information about the kinetics of the photo-cross-linking process, considering that the reaction rate is proportional to the generated heat flow rate [36]. Accordingly, the maxima of photo-DSC thermograms correspond to the time at which maximum photo-cross-linking takes place, which is key information in the printing process. Figure 3 shows the measured heat flow as a function of the irradiation time, and demonstrates that the highest photo-cross-linking rate takes place in around 2 min for AlgMe ink and less than 1 min when additional cross-linking agents are added. Therefore, data evidenced the efficacy of the photo-cross-linking of all the studied inks and their potential for light-mediated printing with curing times less than 2 min for achieving the maximum. As can be observed in Figure 3, the introduction of cross-linking agents in the methacrylated chitosan networks accelerates the photo-curing process enlarging the potential of the mixtures as inks in comparison with pristine AlgMe solution. This fact can be ascribed to the higher functionality and mobility of the cross-linking agents in comparison with the pure methacrylated polymer. These results are in agreement with the gelation times determined by the inverted vial test under the bioprinter conditions, being less than 4 sec in all the samples, which provides practical information for the specific printing process presented here.

**Figure 4:**
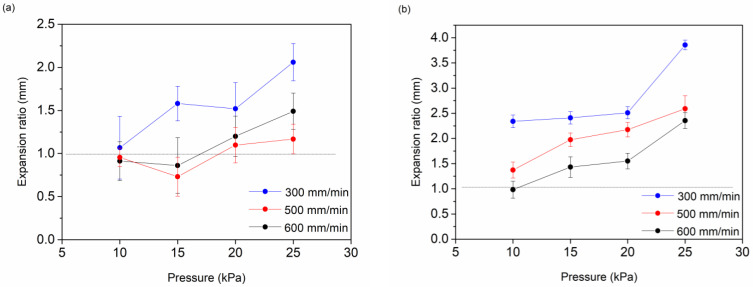
Obtained expansion ratio while using different injection pressures and impression velocity (**a**) using a 0.254 mm nozzle and (**b**) using a 0.41 mm nozzle.

In order to optimize light-mediated extrusion printing, different strands of AlgMe were printed using the bioprinter by varying the cartridge nozzle diameter, impression velocity, and extrusion pressure. As depicted in Figure 4, strands with smaller expansion rates (Equation (3)), i.e., better resolution, were obtained while using the cartridge nozzle with the lowest internal diameter (0.254 mm), due to the lower mass of ink in each deposition. However, despite that, the alginate-based inks employed block easily in the 0.254 mm cartridge nozzle, obtaining poor reproducibility. As shown in Figure 4, as expected, an increase in the impression velocity and a decrease in the pressure resulted in a lower mass of deposited ink, leading consequently to lower expansion ratios. As can be observed, the influence of applied pressure on the expansion ratio is more evident when low deposition velocities are used. According to these results, the 0.41 mm nozzle, injection pressure of 10 kPa, and speed of 600 mm/min were selected as the optimum conditions for the extrusion printing of prepared alginate inks.

**Figure 5:**
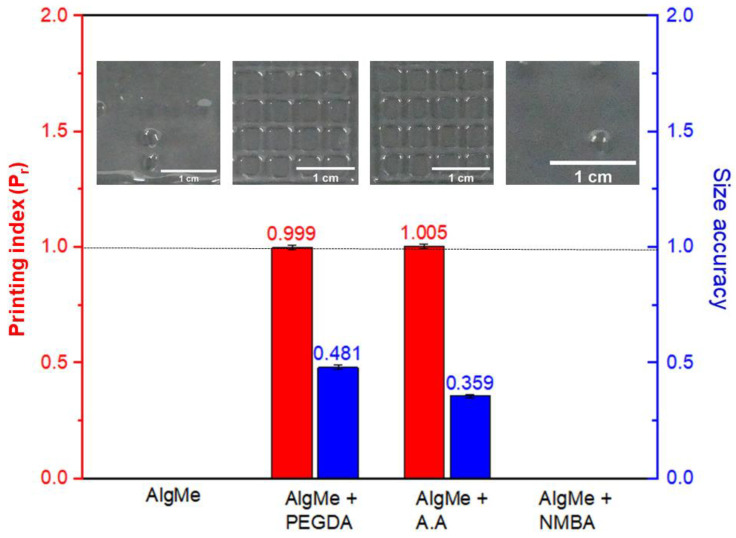
Comparison of the printing quality of the printed square scaffolds of AlgMe without external cross-linking agents (AlgMe) and with PEGDA (AlgMe + PEGDA), acrylic acid (AlgMe + AA), and *N*,*N*’-methylenebisacrylamide (AlgMe + NMBA).

In order to compare the printability of the prepared alginate inks, square scaffolds were printed (0.41 mm, 600 mm/min, and 10 kPa, respectively) and measured size accuracy and printing index parameters (Equations (4) and (5)) were analyzed (Figure 5).

**Figure 6:**
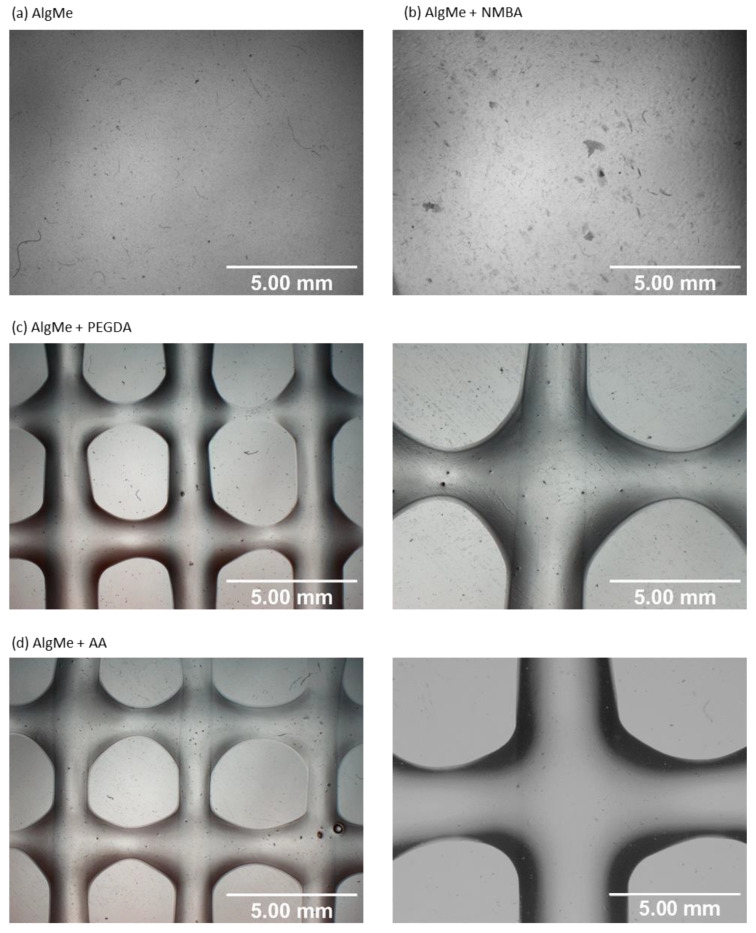
Microscope photographs of the obtained scaffolds: (**a**) AlgMe, (**b**) AlgMe + NMBA, (**c**) AlgMe + PEGDA at 1× and 2×, respectively, and (**d**) AlgMe + AA at 1× and 2×, respectively.

**Figure 7:**
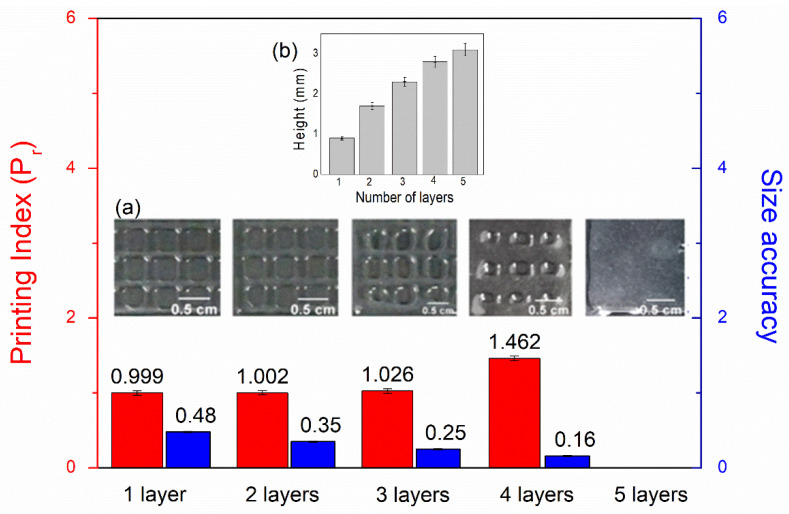
(**a**) Printing index (Pr) and size accuracy parameters while adding a new layer to the printed AlgMe + PEGDA scaffold. (**b**) Effect of the addition of a new layer in obtained height (mm) for AlgMe + PEGDA ink.

As Figure 6 and Figure 7 show, square structures could not be printed in the case of pristine AlgMe ink following specified conditions, and external cross-linking agents were required to achieve adequate printability. The influence of the molecular structure of the employed cross-linker in the printability of the ink is noteworthy. Indeed, the rigid structure of NMBA does not promote the printing of square scaffolds of AlgMe ink, unlike when flexible and long-chain cross-linking agents, such as PEGDA and AA, are included in the formulations. In fact, PEGDA and AA inks lead to high values of the printing index (Pr) (~1) and acceptable size accuracy values (~0.4–0.5). High-quality impressions are obtained by cross-linking with PEGDA, the chains of which react more easily with the acryloyl groups of modified alginate, thus obtaining a more effective cross-linking. Shorter cross-linkers, instead, can bring the polymeric network closer together, making it less flexible [37]. In fact, while NMBA is used, the hydrogel becomes more rigid because the cross-linker is a short molecule [38], thus obtaining poor printability. Acrylic acid monomer is photopolymerized under UV irradiation, forming polyacrylic chains that act as cross-linkers [39]. In this case, neither the flexibility of the hydrogel nor the chain length of the polymerized cross-linker can be controlled; therefore, the AlgMe + AA hydrogel has intermediate printing quality, but closer to that obtained with the flexible PEGDA cross-linker.

Figure 7a shows the printing index (Pr) and size accuracy parameters when one to five layers are printed for AlgMe + PEGDA square structures. The printing index and size accuracy decrease when extra layers are added, the maximum number of layers that allow the obtaining of pores being four. The obtained heights for one to five layers are presented in Figure 7b. Considering that values of 1–10 mm are of suitable thickness for in vitro testing [40], printed structures are adequate to be used for in vitro applications. Due to the lack of printability of the ink based on NMBA, it was discarded for further characterization.

### 3.3. Hydrogel Characterization

**Figure 8:**
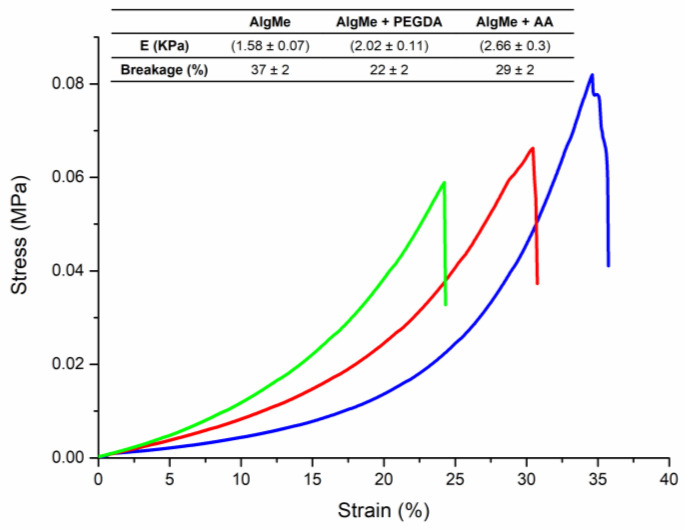
Stress–strain diagram of AlgMe (blue), AlgMe + PEGDA (red), and AlgMe + AA (green) hydrogels and calculated Young’s moduli (12–22% strain).

Figure 8 shows typical compression stress–strain curves measured for the prepared hydrogels. The obtained Young’s moduli were similar to those reported in the bibliography [34]. The collected data reveal that the incorporation of PEGDA and AA as cross-linking agents leads to stiffer and brittle hydrogels, resulting in increased Young’s moduli and decreased breaking strains compared to AlgMe ink, due to the expected increase in cross-linking density [41]. When selected cross-linkers are compared, it can be observed that cross-linking with PEGDA results in a higher breaking strain, which can be ascribed to the flexible nature of the –CH_2_-O- bonds in the backbone of their chains. In addition, samples cross-linked with AA showed higher Young’s moduli, which points to additional interactions between polymerized PAA and the alginate network, such as H-bonds, leading to an interpenetrating network with enhanced mechanical properties.

**Figure 9:**
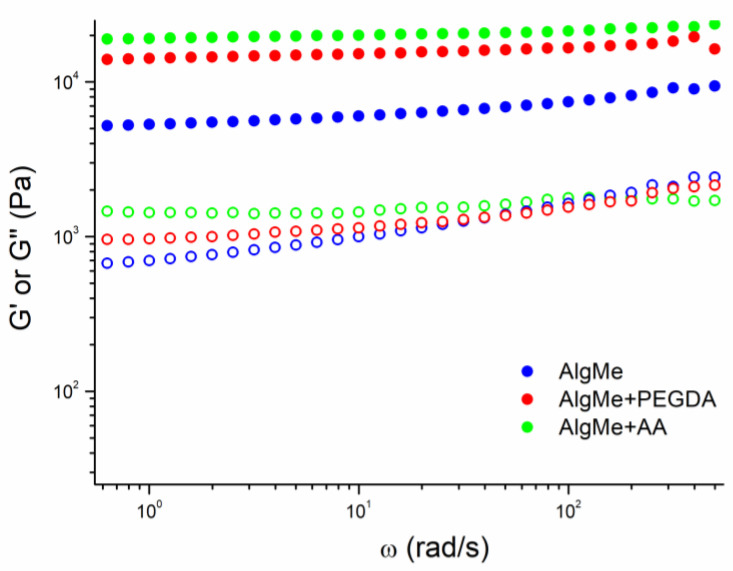
Storage (G’, filled circles) and loss moduli (G’’, open circles) of methacrylated alginate hydrogels (blue) with PEGDA (red) and AA (green) as cross-linking agents (1% strain).

The rheological characteristics of the prepared hydrogels were evaluated by frequency sweep tests at room temperature (Figure 9). All samples showed gel-like behavior with a storage modulus (G’) higher than the loss modulus (G’’) in all frequency ranges. This behavior indicates that the elastic properties of the analyzed materials predominate over the viscous ones, which is behavior typical of hydrogels.

An increase in G’ was observed for the inks with cross-linkers when compared with the ink composed of pure AlgMe, due to the stiffer nature of obtained hydrogels, which is endorsed by the increase in the cross-linking density of the network. These results are in agreement with the analysis of the mechanical properties. The solution of AlgMe + AA also displayed the highest G’ value, which is in line with its interpenetrating network structure.

**Figure 10:**
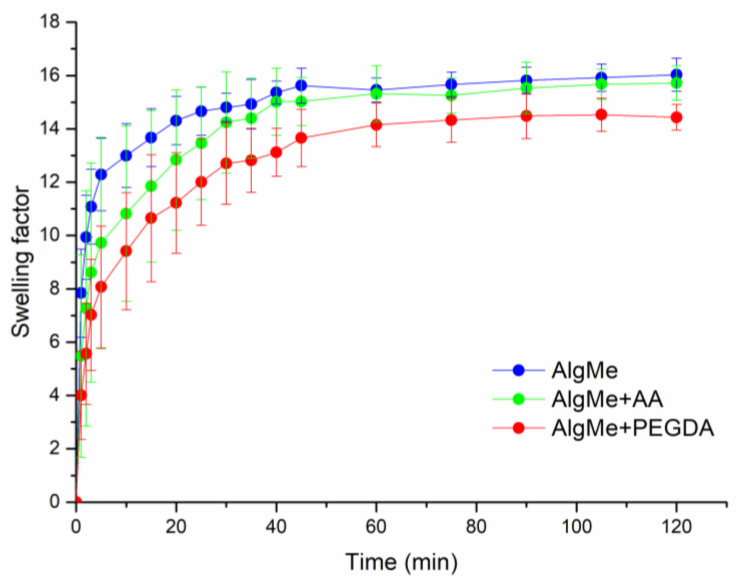
Swelling behavior of the AlgMe (blue) inks while using PEGDA (red) and AA (green) as cross-linkers.

The effect of the incorporation of PEGDA and AA as cross-linking agents in AlgMe ink on the swelling capacity of photo-cross-linked hydrogels was evaluated (Figure 10). As displayed in Figure 10, a similar swelling behavior was observed in PBS at pH = 7.4 in all the samples, despite the differences evidenced in the study of the mechanical and rheological properties of the gels. This fact can be explained by the balance of two opposite effects. On the one hand, the addition of the cross-linking agents leads to a higher cross-linking density that negatively affects the swelling of produced networks. However, on the other hand, the highly hydrophilic nature of incorporated cross-linker molecules favors the swelling of the hydrogels, countering the cross-linking effect. In the case of AA, the high cross-linking density of the interpenetrating networks observed in the rheological and mechanical properties analysis is canceled out by the well-known increased swelling ability of carboxylate polar groups that are ionized at the selected pH values.

**Figure 11:**
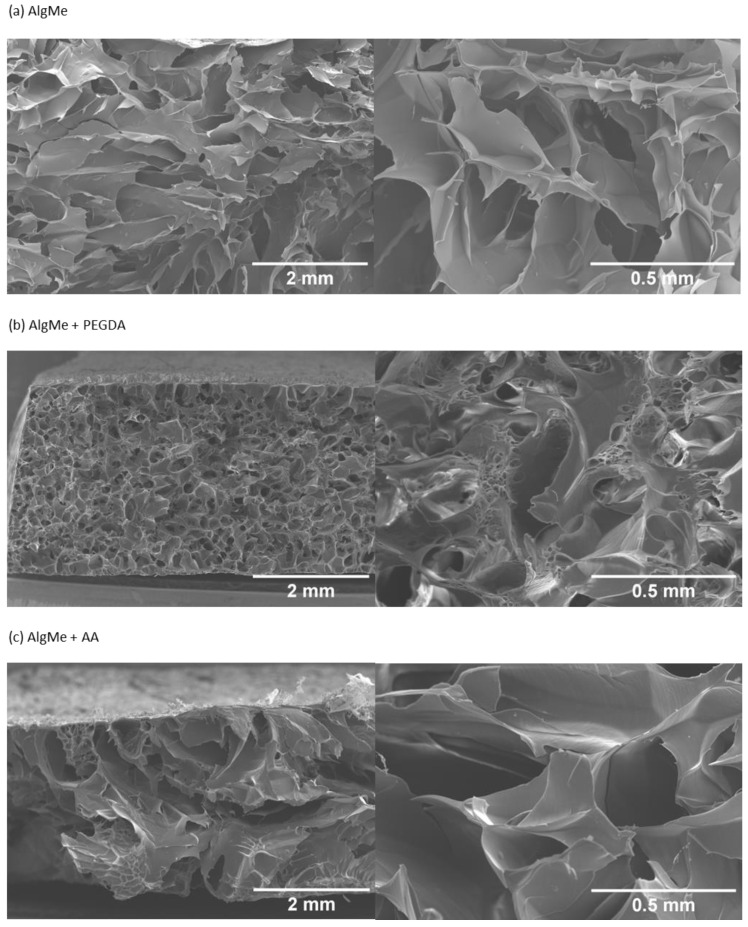
SEM micrographs of the prepared hydrogels without and with both cross-linkers: (**a**) AlgMe, (**b**) AlgMe + PEGDA, and (**c**) AlgMe + AA.

Since pore size is one of the most influential factors in swelling, it was comparatively analyzed by SEM (Figure 11).

The pore size of the hydrogels was analyzed by scanning electron microscopy (SEM), from it was confirmed that the interactions established between polysaccharide chains led to interconnected porous three-dimensional structures. Figure 11 shows the representative SEM micrographs of a cross section of the printed alginate hydrogels. The average pore size in AlgMe is 0.6 ± 0.4 mm, in AlgMe + PEGDA 0.2 ± 0.1 mm, and in AlgMe + AA 0.6 ± 0.3 mm. PEGDA has a known molecular weight and it is smaller than the expected polymerized chains of poly acrylic acid; therefore, a shorter distance between chains is obtained with PEGDA, giving rise to smaller pores. The results obtained of the swelling behavior (Figure 11) are in agreement with these morphological features. The water uptake is smaller for the samples with smaller pores.

### 3.4. In Vitro Degradation

**Figure 12:**
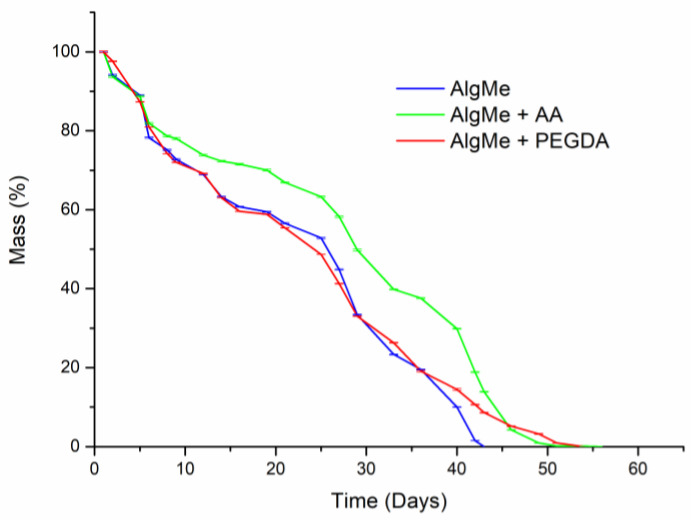
Degradation profiles in hydrolytic media (PBS, pH = 7.4) of the prepared hydrogels: AlgMe (blue), AlgMe + PEGDA (red) and AlgMe + AA (green). Data shown represent the average ± standard deviation of three replicates.

The study of degradation kinetics is a key factor, since it directly affects the stability of the materials and therefore their applicability. The hydrolytic degradation of the hydrogels under physiological conditions (pH = 7.4) is presented in Figure 12. As can be observed, a similar degradation profile was measured regardless of the addition of cross-linking agents, which is in agreement with the similar swelling behavior of the hydrogels. In all the cases, more than 40 days was required to achieve total degradation of the samples, and hydrogel degradation followed second-order degradation kinetics regardless of the cross-linker.

### 3.5. Hydrogel Biocompatibility

**Figure 13:**
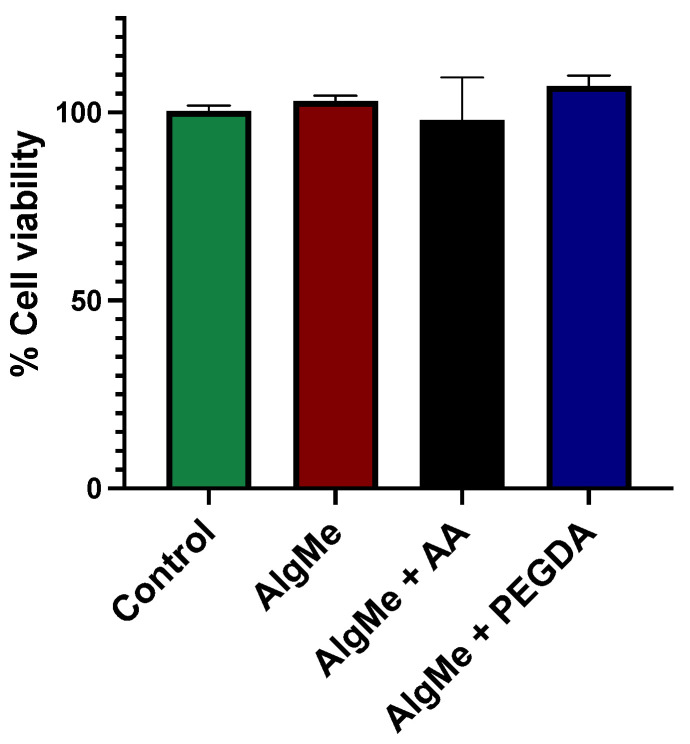
Biocompatibility assessment of hydrogels. Viability of cells cultured with three different hydrogel formulations—AlgMe (red), AlgMe + AA (black), and AlgMe + PEGDA (blue)—in comparison to control cells grown without hydrogel (green). The similarity in cellular viability among the groups confirms the noncytotoxic nature of the hydrogels, indicating their biocompatibility. Data represent mean values ± standard deviations from three independent experiments.

In the context of hydrogel biocompatibility, we quantitatively compared the proliferative activity of HEK293 cultured in 96-well culture plates with or without photo-cross-linked hydrogels for 24 and 48 h, as described in Materials and Methods. Figure 13 shows a quantitative assessment of cellular viability in vitro. The obtained results demonstrated the biocompatibility of the three hydrogels by comparing cellular viability against control cells grown in the absence of hydrogel. This indicates that the hydrogels do not adversely affect cell growth, confirming their biocompatibility in this assay.

## 4. Conclusions

Only ink solutions containing PEGDA and AA were found to be suitable for light-mediated 3D extrusion printing, showing reasonable size accuracy and printing indices in the printing of square basic structures, unlike NMBA-containing inks. The addition of PEGDA and AA as cross-linkers resulted in an improvement in the mechanical and rheological properties of the hydrogels. Specifically, the interpenetrating network developed in the photo-cross-linking of AlgMe + AA ink led to higher stiffness in the gels (2.7 KPa). Despite the differences in the chemical structure of the cross-linking agents employed, similar swelling and degradation behaviors were observed for all the samples. Thus, methacrylated alginate inks containing PEGDA and AA have great potential as printable and nontoxic materials in a wide range of applications in the biomedicine, tissue engineering, and food fields.

## Data Availability

All data and materials are available on request from the corresponding author. The data are not publicly available due to ongoing research using some of the data.

## References

[1] Derakhshanfar S., Mbeleck R., Xu K., Zhang X., Zhong W., Xing M. (2018). 3D Bioprinting for Biomedical Devices and Tissue Engineering: A Review of Recent Trends and Advances. Bioact. Mater..

[2] Mallakpour S., Azadi E., Hussain C.M. (2021). State-of-the-Art of 3D Printing Technology of Alginate-Based Hydrogels—An Emerging Technique for Industrial Applications. Adv. Colloid Interface Sci..

[3] Nachal N., Moses J.A., Karthik P., Anandharamakrishnan C. (2019). Applications of 3D Printing in Food Processing. Food Eng. Rev..

[4] Ozbolat I.T., Peng W., Ozbolat V. (2016). Application Areas of 3D Bioprinting. Drug Discov. Today.

[5] Vanderploeg A., Lee S.E., Mamp M. (2017). The Application of 3D Printing Technology in the Fashion Industry. Int. J. Fash. Des. Technol. Educ..

[6] Zhang X.N., Zheng Q., Wu Z.L. (2022). Recent Advances in 3D Printing of Tough Hydrogels: A Review. Compos. Part B Eng..

[7] Li J., Wu C., Chu P.K., Gelinsky M. (2020). 3D Printing of Hydrogels: Rational Design Strategies and Emerging Biomedical Applications. Mater. Sci. Eng. R Rep..

[8] Xie M., Su J., Zhou S., Li J., Zhang K. (2023). Application of Hydrogels as Three-Dimensional Bioprinting Ink for Tissue Engineering. Gels.

[9] Zhang H., Zhang F., Yuan R. (2019). Applications of Natural Polymer-Based Hydrogels in the Food Industry.

[10] Lee K.Y., Mooney D.J. (2012). Alginate: Properties and Biomedical Applications. Prog. Polym. Sci..

[11] Axpe E., Oyen M.L. (2016). Applications of Alginate-Based Bioinks in 3D Bioprinting. Int. J. Mol. Sci..

[12] Coates E.E., Riggin C.N., Fisher J.P. (2013). Photocrosslinked Alginate with Hyaluronic Acid Hydrogels as Vehicles for Mesenchymal Stem Cell Encapsulation and Chondrogenesis. J. Biomed. Mater. Res.-Part A.

[13] Chiulan I., Heggset E.B., Voicu Ş.I., Chinga-Carrasco G. (2021). Photopolymerization of Bio-Based Polymers in a Biomedical Engineering Perspective. Biomacromolecules.

[14] Gao Y., Jin X. (2019). Dual Crosslinked Methacrylated Alginate Hydrogel Micron Fibers and Tissue Constructs for Cell Biology. Mar. Drugs.

[15] Jeong S.I., Jeon O., Krebs M.D., Hill M.C., Alsberg E. (2013). Biodegradable Photo-Crosslinked Alginate Nanofibre Scaffolds with Tuneable Physical Properties, Cell Adhesivity and Growth Factor Release. Eur. Cells Mater..

[16] Mignon A., Devisscher D., Graulus G.J., Stubbe B., Martins J., Dubruel P., De Belie N., Van Vlierberghe S. (2017). Combinatory Approach of Methacrylated Alginate and Acid Monomers for Concrete Applications. Carbohydr. Polym..

[17] Mishbak H.H., Cooper G., Bartolo P.J. (2019). Development and Characterization of a Photocurable Alginate Bioink for Three-Dimensional Bioprinting. Int. J. Bioprint..

[18] Mistry P., Aied A., Alexander M., Shakesheff K., Bennett A., Yang J. (2017). Bioprinting Using Mechanically Robust Core–Shell Cell-Laden Hydrogel Strands. Macromol. Biosci..

[19] Hou S., Ma P.X. (2015). Stimuli-Responsive Supramolecular Hydrogels with High Extensibility and Fast Self-Healing via Precoordinated Mussel-Inspired Chemistry. Chem. Mater..

[20] Hu J., Hou Y., Park H., Choi B., Hou S., Chung A., Lee M. (2012). Visible Light Crosslinkable Chitosan Hydrogels for Tissue Engineering. Acta Biomater..

[21] Kunwar P., Jannini A.V.S., Xiong Z., Ransbottom M.J., Perkins J.S., Henderson J.H., Hasenwinkel J.M., Soman P. (2020). High-Resolution 3D Printing of Stretchable Hydrogel Structures Using Optical Projection Lithography. ACS Appl. Mater. Interfaces.

[22] Monteiro N., Thrivikraman G., Athirasala A., Tahayeri A., França C.M., Ferracane J.L., Bertassoni L.E. (2018). Photopolymerization of Cell-Laden Gelatin Methacryloyl Hydrogels Using a Dental Curing Light for Regenerative Dentistry. Dent. Mater..

[23] Ooi H.W., Mota C., Tessa Ten Cate A., Calore A., Moroni L., Baker M.B. (2018). Thiol-Ene Alginate Hydrogels as Versatile Bioinks for Bioprinting. Biomacromolecules.

[24] Rouillard A.D., Berglund C.M., Lee J.Y., Polacheck W.J., Tsui Y., Bonassar L.J., Kirby B.J. (2011). Methods for Photocrosslinking Alginate Hydrogel Scaffolds with High Cell Viability. Tissue Eng.-Part C Methods.

[25] Zhao D., Tie C., Cheng B., Yang S., Wang X., Sun Z., Yin M., Zhu H., Yin M. (2020). Effect of Altering Photocrosslinking Conditions on the Physical Properties of Alginate Gels and the Survival of Photoencapsulated Cells. Polym. Degrad. Stab..

[26] Lewandowska-Łańcucka J., Mystek K., Mignon A., Van Vlierberghe S., Łatkiewicz A., Nowakowska M. (2017). Alginate- and Gelatin-Based Bioactive Photocross-Linkable Hybrid Materials for Bone Tissue Engineering. Carbohydr. Polym..

[27] Hasany M., Talebian S., Sadat S., Ranjbar N., Mehrali M., Wallace G.G., Mehrali M. (2021). Synthesis, Properties, and Biomedical Applications of Alginate Methacrylate (ALMA)-Based Hydrogels: Current Advances and Challenges. Appl. Mater. Today.

[28] Jeon O., Bouhadir K.H., Mansour J.M., Alsberg E. (2009). Photocrosslinked Alginate Hydrogels with Tunable Biodegradation Rates and Mechanical Properties. Biomaterials.

[29] Hamedi E., Vahedi N., Sigaroodi F., Parandakh A., Hosseinzadeh S., Zeinali F., Khani M.M. (2023). Recent Progress of Bio-Printed PEGDA-Based Bioinks for Tissue Regeneration. Polym. Adv. Technol..

[30] Cha C., Kim S.Y., Cao L., Kong H. (2010). Decoupled Control of Stiffness and Permeability with a Cell-Encapsulating Poly(Ethylene Glycol) Dimethacrylate Hydrogel. Biomaterials.

[31] Bekin S., Sarmad S., Gürkan K., Keçeli G., Gürdaǧ G. (2014). Synthesis, characterization and bending behavior of electroresponsive sodium alginate/poly(acrylic acid) interpenetrating network films under an electric field stimulus. Sens. Actuators B.

[32] Chou A.I., Nicoll S.B. (2009). Characterization of Photocrosslinked Alginate Hydrogels for Nucleus Pulposus Cell Encapsulation. J. Biomed. Mater. Res.-Part A.

[33] Araiza-verduzco F., Rodr E., Cruz H., Alatorre-meda M. (2019). Photocrosslinked Alginate-Methacrylate Hydrogels with Modulable Mechanical Properties: Effect of the Molecular Conformation and Electron Density of the Methacrylate Reactive Group. Materials.

[34] Mignona A., Devisscherb D., Graulusb G.J., Stubbeb B., Martins J., Dubruelb P., De Beliea N., Van Vlierbergheb S. (2017). Combinatory approach of methacrylated alginate and acid monomersfor concrete applications. Carbohydr. Polym..

[35] Ouyang L., Yao R., Zhao Y., Sun W. (2016). Effect of Bioink Properties on Printability and Cell Viability for 3D Bioplotting of Embryonic Stem Cells. Biofabrication.

[36] Rusu M.C., Block C., Van Assche G., Van Mele B. (2012). Influence of Temperature and UV Intensity on Photo-Polymerization Reaction Studied by Photo-DSC. J. Therm. Anal. Calorim..

[37] Poursamar S.A., Lehner A.N., Azami M., Ebrahimi-Barough S., Samadikuchaksaraei A., Antunes A.P.M. (2016). The Effects of Crosslinkers on Physical, Mechanical, and Cytotoxic Properties of Gelatin Sponge Prepared via in-Situ Gas Foaming Method as a Tissue Engineering Scaffold. Mater. Sci. Eng. C.

[38] Maitra J., Shukla V.K. (2014). Cross-Linking in Hydrogels—A Review. Am. J. Polym. Sci..

[39] Wang H., Brown H.R. (2004). Self-Initiated Photopolymerization and Photografting of Acrylic Monomers. Macromol. Rapid Commun..

[40] Jakus A.E., Rutz A.L., Shah R.N. (2016). Advancing the Field of 3D Biomaterial Printing. Biomed. Mater..

[41] Sridharan R., Ryan E.J., Kearney C.J., Kelly D.J., O’Brien F.J. (2019). Macrophage Polarization in Response to Collagen Scaffold Stiffness Is Dependent on Cross-Linking Agent Used to Modulate the Stiffness. ACS Biomater. Sci. Eng..

